# White matter lesion effect modification of aspirin and unfractionated heparin during endovascular stroke treatment

**DOI:** 10.1186/s13244-025-02095-2

**Published:** 2025-10-22

**Authors:** Laura M. van Poppel, Lucas de Vries, Mahsa Mojtahedi, Henk van Voorst, Praneeta R. Konduri, Rob A. van de Graaf, Wouter van der Steen, Laura Martou, Paul Bentley, Henk A. Marquering, Bart J. Emmer, Charles B. L. M. Majoie

**Affiliations:** 1 Department of Radiology and Nuclear Medicine, Amsterdam UMC, Amsterdam, The Netherlands; 2 Department of Biomedical Engineering and Physics, Amsterdam UMC, Amsterdam, The Netherlands; 3 Amsterdam Cardiovascular Sciences, Amsterdam, The Netherlands; 4 Amsterdam Neuroscience, Amsterdam, The Netherlands; 5 Informatics Institute, University of Amsterdam, Amsterdam, The Netherlands; 6 Department of Radiology, Stanford University School of Medicine, Palo Alto, CA USA; 7 Department of Radiology and Nuclear Medicine, Erasmus MC University Medical Center, Rotterdam, The Netherlands; 8 Department of Neurology, Erasmus MC University Medical Center, Rotterdam, The Netherlands; 9 Department of Brain Sciences, Imperial College London, London, UK

**Keywords:** Ischemic stroke, Leukoencephalopathies, Endovascular procedures, Tomography (X-ray computed), Deep learning

## Abstract

**Objectives:**

Periprocedural aspirin or unfractionated heparin during endovascular treatment in acute ischemic stroke increases symptomatic intracranial hemorrhage (sICH) risk without improving functional outcome. White matter lesions (WMLs) are associated with higher sICH risk and poor functional outcome following stroke. We aimed to assess whether WML volume modifies the effect of aspirin or heparin.

**Materials and methods:**

In this post-hoc analysis of the MR CLEAN-MED trial, WML volume was automatically determined using deep learning-based segmentation on baseline non-contrast CT scans. Outcomes included good functional outcome (modified Rankin Scale 0–2 at 90 days), any ICH, asymptomatic ICH (aICH), and sICH. Patients received either aspirin or not, and either heparin or not. Multivariable logistic regression evaluated treatment effect and effect modification.

**Results:**

Of 628 patients, 614 with baseline CT were included. Median WML volume was 0.59 mL without significant differences between treatment arms. WML volume significantly modified the effect of aspirin on sICH (*p* = 0.01), but not on functional outcome (*p* = 0.95), any ICH (*p* = 0.52), or aICH (*p* = 0.30). Aspirin was associated with increased sICH risk, which decreased with increasing WML volume (aOR 0.96 [95% CI: 0.93–0.99] per 1 mL). For patients with large WML volumes, aspirin showed no significant effect on sICH risk. The effect of heparin on functional outcome, any ICH, aICH, and sICH was not modified by WML volume (*p* = 0.53, *p* = 0.26, *p* = 0.08, *p* = 0.63, respectively).

**Conclusions:**

WML volume significantly modified the effect of aspirin on sICH risk, with aspirin-associated risk decreasing as WML volume increased. WML volume did not modify the effect of aspirin or heparin on other outcomes.

**Critical relevance statement:**

WML volume on non-contrast CT modifies the effect of aspirin during endovascular thrombectomy on sICH risk, yet no WML-based patient subgroup showed save benefits from periprocedural aspirin or heparin treatment.

**Key Points:**

- Periprocedural aspirin and unfractionated heparin during endovascular treatment cause a higher hemorrhage risk.
- WML volume is associated with worse functional outcome and WML volume significantly modifies the effect of aspirin on symptomatic hemorrhage risk, with aspirin-associated risk decreasing with increasing WML volume.
- No WML-volume-based patient subgroup was identified where aspirin or heparin treatment demonstrated safe clinical benefit.

**Graphical Abstract:**

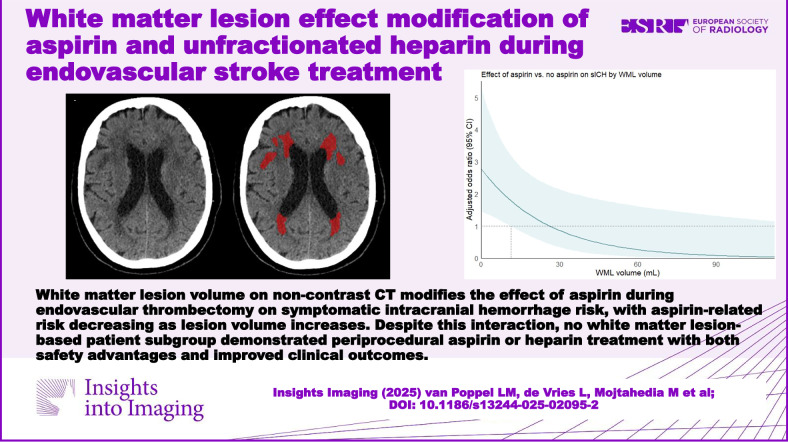

## Introduction

White matter lesions (WMLs), indicative of small vessel disease in the brain, are often observed in patients with an acute ischemic stroke (AIS) due to an anterior circulation large vessel occlusion (LVO) [1, 2]. Prior systematic reviews and meta-analyses have linked an increased WML burden to a higher likelihood of symptomatic intracranial hemorrhage (sICH) and poor functional outcome following intravenous thrombolysis (IVT) [3, 4], and poor functional outcome following endovascular thrombectomy (EVT) [5].

The MR CLEAN-MED trial demonstrated that periprocedural intravenous aspirin and heparin during EVT increase sICH risk without improving functional outcomes in the overall population [6]. However, specific patient subgroups may have differential risk-benefit profiles based on underlying pathophysiology. Given that both increased WML burden and periprocedural treatment with aspirin and heparin are associated with an elevated risk of sICH, this raises the possibility of individualized treatment effects based on WML burden. Understanding how WML burden modifies these treatment effects could inform personalized approaches in scenarios requiring therapy with aspirin or heparin, such as acute stenting procedures or rescue medication.

Assessment of WML burden is challenging, especially in the acute setting, where non-contrast computed tomography (NCCT) is the predominant imaging modality. WMLs’ visibility on NCCT is limited. The conventional grading scales, such as the Van Swieten scale and Fazekas score, have been developed for MRI assessment and suffer from interobserver variability [7]. Alternatively, our previously developed automated NCCT-based WML segmentation model offers the possibility to extract WML volume from NCCT, providing a quantitative measure of WML burden [8].

We hypothesize that WML volume modifies the observed effect of heparin and aspirin administered during EVT in AIS patients due to an anterior circulation LVO. Specifically, we hypothesize that patients with large WML volumes, likely to suffer from WML-associated pathological mechanisms and risk factors, are at an increased risk of worse outcomes and hemorrhages when allocated to receive aspirin or heparin, while those with smaller WML volumes are not.

## Materials and methods

### Data and study design

In this post hoc analysis, we included data of all patients with a baseline NCCT scan from the MR CLEAN (Multicenter Randomized Clinical Trial of Endovascular Treatment for Acute Ischemic Stroke in the Netherlands) MED study: a multicenter trial aiming to assess the safety and efficacy of intravenous aspirin and unfractionated heparin, started during EVT in AIS patients with an anterior circulation LVO [6]. Specifically, patients were randomized in a factorial design to receive aspirin or no aspirin, and heparin or no heparin, resulting in four possible treatment groups: aspirin only, heparin only, both aspirin and heparin, or neither (control). The MR CLEAN-MED trial was conducted with the approval of a central medical ethics committee [9] and the research board of each participating center. All centers used a deferred consent procedure in accordance with national legislation. All patients or their legal representatives provided written deferred consent after randomization and study treatment. We have followed the STROBE guidelines and completed a STROBE checklist (Appendix 1). To assess whether the observed effect of aspirin and heparin treatment in the trial was modified by WMLs, we used the same study population: the modified intention-to-treat population, consisting of all patients for whom consent was obtained.

### Assessment of WML characteristics

WMLs were automatically segmented from baseline NCCT using an improved version of our previously validated nnU-Net model [8]. The model was trained using 100 NCCTs with WML annotations from three expert annotators that were all used as ground truth labels, incorporating inter-rater variability in model training. An additional 50 NCCTs from patients without WMLs were included in the training set, allowing the model to learn the appearance of healthy brain tissue and improve specificity by reducing false positive segmentations. The model was tested on a separate test set against the expert annotations. WML volume was then calculated from these automated segmentations (detailed methodology in Appendix 2).

### Statistical analysis

The primary outcome was a good functional outcome, defined as a score of 0–2 on the modified Rankin Scale (mRS) at 90 days. Safety outcomes included any ICH, and its subdivision into aICH and sICH, according to the Heidelberg Bleeding Classification [10].

We conducted all analyses separately for aspirin allocation (aspirin vs no aspirin) and heparin allocation (heparin vs no heparin), analyzing each medication independently given its distinct pharmacodynamic mechanisms. This approach is consistent with the original trial, which assumed independence of effects and confirmed this assumption with interaction analyses [6].

Following the recommendations for effect modification analysis [11], we employed a two-step analytical approach: First, we evaluated both the independent effect of treatment allocation on outcomes and the independent association of WML volume with outcomes, using additive logistic regression models. Second, we included a multiplicative interaction term between WML volume and treatment allocation to assess effect modification, examining treatment effects within the context of varying WML volumes. All models were fitted both unadjusted and adjusted for confounders. We compared the additive and multiplicative interaction models using a likelihood ratio test, considering *p* ≤ 0.05 as statistically significant for effect modification.

In line with the MR CLEAN-MED trial, all analyses were adjusted for the following baseline prognostic variables: age, pre-stroke mRS score, time from onset to door of endovascular treatment center, time from door of endovascular treatment center to groin puncture, National Institutes of Health Stroke Scale (NIHSS) score, collateral score, and inclusion before or after early termination of moderate-dose unfractionated heparin arms. For the additive models, we report the unadjusted and adjusted odds ratios (OR and aOR, respectively) with corresponding 95% confidence intervals (CIs) of WML volume and treatment allocation. For the interaction models, we present the treatment effect in patients without WMLs (the reference category) and the difference in its treatment effectiveness for every 1 mL increase in WML volume (the interaction term), again using (adjusted) odds ratios.

If significant effect modification was shown, we plotted the change in the aOR and 95% CI across the range of WML volume calculated from the multiplicative interaction model. We visually identified the point where the lower bound of the 95% CI crossed 1.0, indicating the WML volume threshold where the effect of the treatment was no longer statistically significant. We performed two sensitivity analyses, additionally adjusting for (i) treatment with IVT, and (ii) prior use of antithrombotics, including prior use of antiplatelets, vitamin K antagonists, or DOACs, as defined by the MR CLEAN-MED trial. Missing values were replaced using multiple imputations (*n* = 5, aregImpute function, 8 iterations). All statistical analyses were performed using R (version 3.6.3).

## Results

### Patient characteristics

We included data from 614 of the 628 patients from the MR CLEAN-MED trial. Of these 614 patients, 303 (49%) were allocated to receive aspirin, 311 (51%) to no aspirin, 326 (53%) to receive unfractionated heparin, and 288 (47%) to no unfractionated heparin. Baseline characteristics and process measures per treatment allocation have been previously published in the main trial paper [6]. The median WML volume was 0.59 mL (interquartile range, 0.05–5.60 mL). The WML volume was not significantly different between the group allocated to receive aspirin and no aspirin (Wilcoxon test, *p* = 0.88) and allocated to receive heparin and no heparin (Wilcoxon test, *p* = 0.34).

**Fig. 1:**
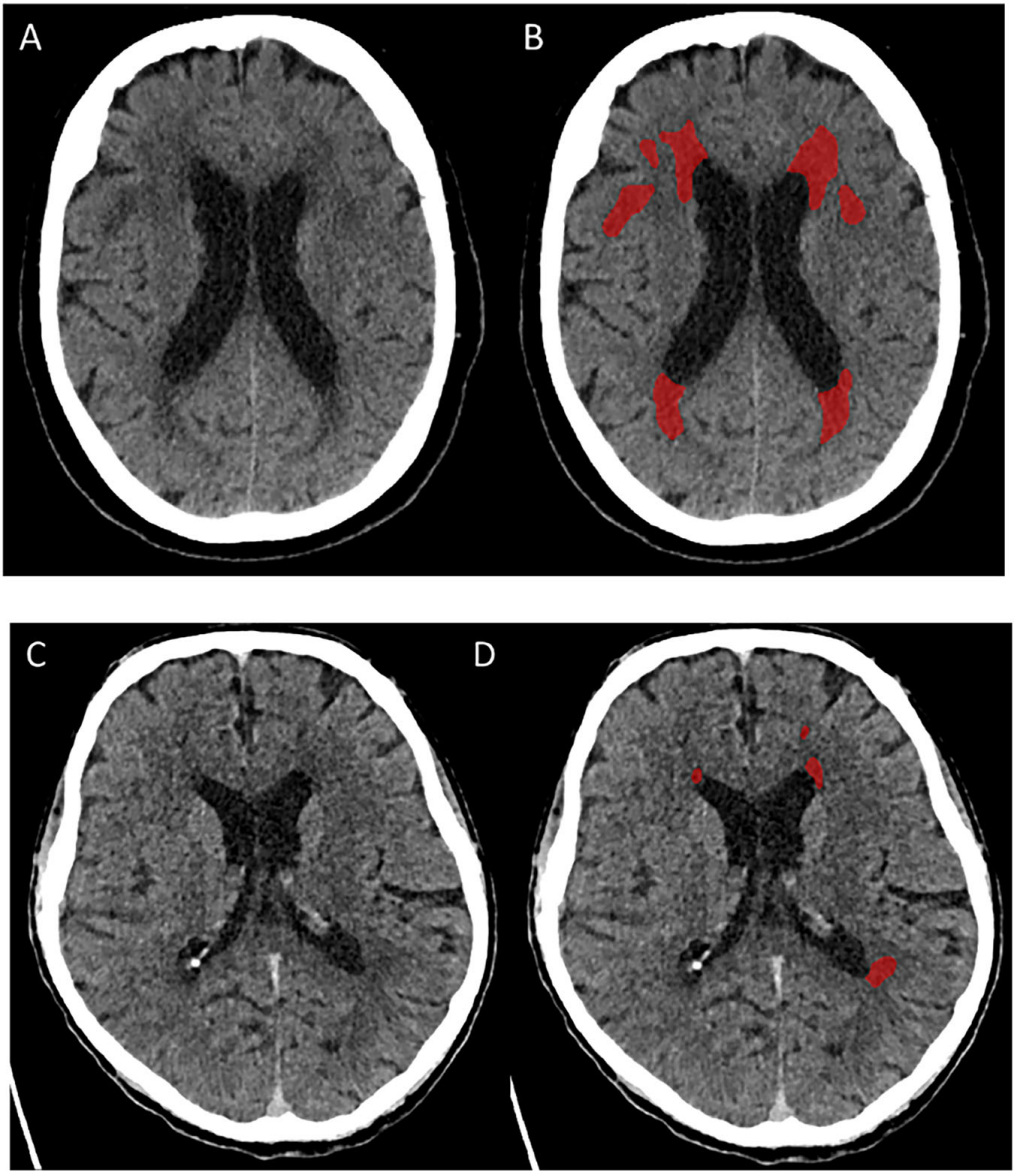
WML segmentation examples for two patients in the MR CLEAN-MED trial. Left: NCCT (**A**) with a WML segmentation in red (**B**) with a total volume of 48 mL. Right: NCCT (**C**) with a WML segmentation in red (**D**) with a total volume of 2.6 mL

Figure 1 shows examples of patients’ WML segmentation and calculated WML volumes. Baseline demographic, clinical, and outcome variables, categorized per tertile of WML volume, were compared with standard statistics (Table 1). Patients with larger WML volumes were older (*p* < 0.001), had higher pre-stroke mRS scores (*p* < 0.001), higher baseline Alberta Stroke Program Early CT score (ASPECTS) (*p* < 0.001), and more often had a history of atrial fibrillation (*p* < 0.001), hypertension (*p* < 0.001), ischemic stroke (*p* < 0.001), and antithrombotic use (*p* < 0.001). Distributions of occlusion location differed between subgroups (*p* = 0.04), where patients in tertile 1 had relatively more occlusions in the internal carotid artery and less in the M2 segment compared to ones in tertile 2 and 3. Tertile 2 included more males than females, whereas the male-female ratio was balanced in the other two tertiles (*p* = 0.02). Tertile 3 had a longer time from stroke onset to the door of the EVT center (*p* = 0.04). As for outcomes, the rate of good functional outcome decreased with increasing tertile (*p* < 0.001). The incidences of any ICH and aICH were not different between tertiles (*p* = 0.33 and *p* = 0.59, respectively), but sICH occurred more frequently in tertile 3 (*p* = 0.007).

**Table 1:** Baseline characteristics, measures, and outcomes according to WML volume subgroup

	WML volume tertile 1 < 0.1 mL (*n* = 205)	WML volume tertile 2 0.1–2.8 mL (*n* = 204)	WML volume tertile 3 ≥ 2.8 mL (*n* = 205)	*p*-value*
Treatment allocation				
Aspirin	100 (49%)	108 (53%)	95 (46%)	0.40
Heparin	102 (50%)	115 (56%)	109 (53%)	0.41
Age, years	66 (57–74)	72 (65–79)	80 (73–86)	< 0.001
Sex, male	103 (50%)	125 (61%)	99 (48%)	0.02
Transferred from primary hospital	162 (79%)	158 (77%)	168 (83%)	0.52
NIHSS score	15 (8–19)	15 (9–19)	16 (10–20)	0.07
History of atrial fibrillation (missing *n* = 1 (< 1%))	28 (14%)	43 (21%)	82 (40%)	< 0.001
History of hypertension	69 (34%)	107 (53%)	107 (53%)	< 0.001
Previous ischemic stroke (missing *n* = 2 (< 1%))	16 (8%)	35 (17%)	59 (29%)	< 0.001
Pre-stroke mRS score				< 0.001
0	162 (79%)	143 (71%)	104 (52%)	
1	34 (17%)	37 (18%)	43 (21%)	
2	7 (3%)	18 (9%)	34 (17%)	
≥ 3	1 (1%)	3 (2%)	21 (10%)	
Prior use of antithrombotics	72 (35%)	99 (49%)	133 (65%)	< 0.001
Treatment with intravenous thrombolytics	163 (80%)	148 (73%)	143 (70%)	0.07
Time from stroke onset to intravenous thrombolytics, min, (missing *n* = 160 (< 26%))	80 (60–125)	75 (95–101)	83 (60–130)	0.16
ASPECTS (missing *n* = 3 (< 1%))	9 (7–10)	9 (8–10)	10 (8–10)	< 0.001
Occlusion side				0.64
Right hemisphere	102 (50%)	104 (51%)	97 (47%)	
Left hemisphere	101 (49%)	100 (49%)	107 (52%)	
No occlusion visible	2 (1%)		1 (1%)	
Occluded segment (missing *n* = 3 (<1%))				0.04
Internal carotid artery	49 (24%)	34 (17%)	28 (14%)	
Terminal internal carotid artery	21 (10%)	16 (8%)	14 (7%)	
Middle cerebral artery M1 segment	94 (46%)	102 (50%)	104 (51%)	
Middle cerebral artery M2 segment	36 (18%)	50 (25%)	57 (28%)	
None	4 (2%)	1	1	
Extracranial internal carotid artery occlusion	18 (9%)	34 (17%)	38 (19%)	0.24
Poor collateral score (< 50%) (missing *n* = 8 (< 1%))	64 (31%)	67 (33%)	81 (40%)	0.17
Time from stroke onset to door of endovascular therapy center, min, (missing *n* = 1 (< 1%))	138 (110–210)	138 (100–181)	152 (114–208)	0.04
Time from the door of the endovascular therapy center to groin puncture, min, (missing *n* = 30 (50%))	33 (22–51)	31 (24–45)	30 (22–50)	0.84
mRS score of 0–2 at 90 days	133 (65%)	114 (56%)	62 (30%)	< 0.001
Any intracranial hemorrhage (missing *n* = 73 (12%))	81 (45%)	77 (42%)	89 (50%)	0.33
aICH (missing *n* = 73 (12%))	66 (36%)	60 (33%)	56 (31%)	0.59
sICH	15 (7%)	17 (8%)	33 (16%)	0.007

Data are median (IQR) or *n* (%)

*NIHSS* National Institutes of Health Stroke Scale [34], *mRS* modified Rankin Scale [35], *ASPECTS* Alberta Stroke Program Early CT score [36]

* *p*-value is calculated using the Kruskal–Wallis test for numerical data and the Chi-square test for categorical data

### Aspirin treatment effect modification by WML volume

In the additive models, patients allocated to receive aspirin had significantly higher odds of sICH (aOR 1.91 [95% CI: 1.1–3.31]), but not of good functional outcome (0.80 [0.55–1.16]), any ICH (1.36 [0.98–1.90]), and aICH (1.07 [0.76–1.51]). WML volume was significantly associated with lower odds of good functional outcome (aOR 0.96 [95% CI: 0.94–0.98]), but not with any ICH (1.00 [0.99–1.01]), aICH (1.00 [0.98–1.01]), and sICH (1.01 [0.99–1.02]) (Table 2).

**Table 2:** Treatment effects with 95% CI derived from the additive and multiplicative interaction models

		Additive model		Multiplicative interaction model		
Outcome		Aspirin	WML volume	Aspirin		*p*-value
		OR (95% CI)	OR (95% CI)	OR (95% CI) WML volume = 0	OR (95% CI) interaction term	Interaction
mRS score of 0–2 at 90 days	Unadjusted	0.78 (0.57–1.07)	0.94 (0.92–0.96)	0.77 (0.53–1.12)	0.99 (0.95–1.03)	0.64
	Adjusted*	0.80 (0.55–1.16)	0.96 (0.94–0.98)	0.79 (0.52–1.20)	1.00 (0.96–1.04)	0.95
Any ICH	Unadjusted	1.35 (0.98–1.85)	1.01 (1.00–1.02)	1.36 (0.96–1.94)	1.00 (0.98–1.02)	0.91
	Adjusted*	1.36 (0.98–1.90)	1.00 (0.99–1.01)	1.43 (0.99–2.07)	0.99 (0.97–1.02)	0.52
aICH	Unadjusted	1.04 (0.75–1.45)	1.00 (0.99–1.01)	0.92 (0.64–1.33)	1.02 (1.00–1.04)	0.12
	Adjusted*	1.07 (0.76–1.51)	1.00 (0.98–1.01)	0.98 (0.67–1.44)	1.01 (0.99–1.04)	0.30
sICH	Unadjusted	2.01 (1.18–3.44)	1.01 (1.00–1.03)	2.95 (1.57–5.52)	0.96 (0.93–0.99)	0.01
	Adjusted*	1.91 (1.1–3.31)	1.01 (0.99–1.02)	2.79 (1.46–5.32)	0.96 (0.93–0.99)	0.01

* Values were adjusted for age, pre-stroke mRS score, NIHSS at baseline, collateral score at baseline, time from onset to door of intervention hospital, time from door intervention hospital to groin puncture, and inclusion before or after early termination of moderate-dose unfractionated heparin arms

*mRS* modified Rankin scale, *(a)ICH or (s)ICH* (asymptomatic or symptomatic) intracranial hemorrhage, *WML* white matter lesion, *OR* odds ratio

**Fig. 2:**
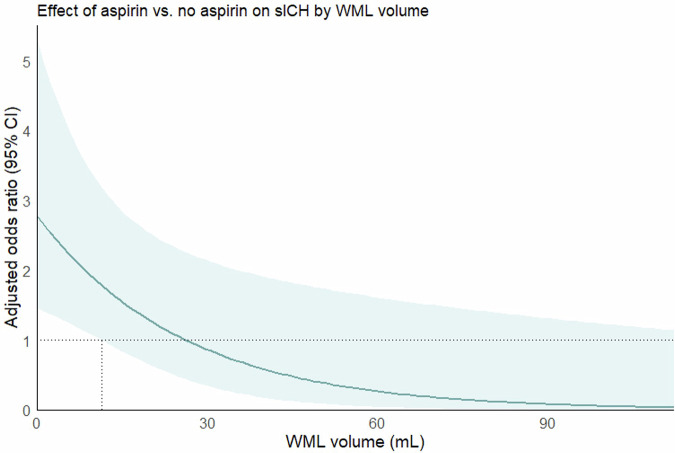
Adjusted odds ratio (95% CI) on sICH for the allocation to receive aspirin compared to no aspirin by WML volume. The adjusted odds ratio (dark blue solid line) and the corresponding 95% CI (light blue shaded area) for the allocation to receive aspirin compared to no aspirin across the entire range of WML volume are plotted. The lower bound of the 95% CI crossed the 1.0 line (dotted horizontal line) at 11.35 mL (dotted vertical line), indicating that aspirin was significantly associated with higher odds of sICH with a WML volume ≤ 11.35 mL. With WML volumes > 11.35 mL, the association became non-significant

Multiplicative interaction analysis showed that WML volume did not significantly modify the effect of aspirin on good functional outcome (*p* = 0.95), any ICH (*p* = 0.52), and aICH (*p* = 0.30). However, the effect of aspirin on sICH was significantly modified by WML volume (*p* = 0.01): in patients without WMLs (volume = 0 mL), there was a significant risk of sICH associated with aspirin (aOR 2.79 [95% CI: 1.46–5.32]). This aspirin-induced sICH risk decreased by 4% for every 1 mL increase in WML volume (0.96 [0.93–0.99]) (Table 2). In the plot that visualizes the aOR of the effect of aspirin on sICH across the entire range of WML volumes (Fig. 2), we can identify that the effect of aspirin became statistically insignificant at WML volumes larger than 11.35 mL, where the lower bound of the 95% CI crossed 1.0.

Both the sensitivity analyses adjusted for treatment with IVT and the one adjusted for prior use of antithrombotics showed similar results as the main analysis (Appendix 3 and 4).

### Heparin treatment effect modification by WML volume

In the additive models, patients allocated to receive heparin had significantly higher odds of sICH (aOR 1.84 [95% CI: 1.05–3.21]), but not of good functional outcome (0.78 [0.53–1.13]), any ICH (1.11 [0.79–1.55]), and aICH (0.87 [0.61–1.23]). WML volume was significantly associated with lower odds of good functional outcome (aOR 0.96 [95% CI: 0.94–0.98]), but not with any ICH (1.00 [0.99–1.01]), aICH (1.00 [0.98–1.01]), and sICH (1.00 [0.99–1.02]) (Table 3).

**Table 3:** Treatment effects with (95% CI) derived from the additive and multiplicative interaction models

		Additive model		Multiplicative interaction model		
Outcome		Heparin	WML volume	Heparin		*p*-value
		OR (95% CI)	OR (95% CI)	OR (95% CI) WML volume = 0	OR (95% CI) interaction term	Interaction
mRS score of 0-2 at 90 days	Unadjusted	0.73 (0.53–1.00)	0.94 (0.92–0.96)	0.83 (0.57–1.20)	0.96 (0.92–1.01)	0.12
	Adjusted*	0.78 (0.53–1.13)	0.96 (0.94–0.98)	0.81 (0.53–1.22)	0.99 (0.95–1.03)	0.65
Any ICH	Unadjusted	1.19 (0.87–1.64)	1.01 (1.00–1.02)	1.24 (0.87–1.76)	0.99 (0.97–1.02)	0.58
	Adjusted*	1.11 (0.79–1.55)	1.00 (0.99–1.01)	1.21 (0.84–1.74)	0.99 (0.96–1.01)	0.26
aICH	Unadjusted	0.95 (0.68–1.32)	1.00 (0.99–1.01)	1.06 (0.73–1.53)	0.98 (0.96–1.01)	0.18
	Adjusted*	0.87 (0.61–1.23)	1.00 (0.98–1.01)	1.00 (0.68–1.47)	0.98 (0.95–1.00)	0.08
sICH	Unadjusted	1.84 (1.07–3.15)	1.01 (1.00–1.03)	1.63 (0.89–2.98)	1.01 (0.98–1.05)	0.47
	Adjusted*	1.84 (1.05–3.21)	1.00 (0.99–1.02)	1.72 (0.92–3.20)	1.01 (0.97–1.05)	0.63

* Values were adjusted for age, pre-stroke mRS score, NIHSS at baseline, collateral score at baseline, time from onset to door of intervention hospital, time from door intervention hospital to groin puncture, and inclusion before or after early termination of moderate-dose unfractionated heparin arms

*mRS* modified Rankin scale, *(a)ICH or (s)ICH* (asymptomatic or symptomatic) intracranial hemorrhage, *WML* white matter lesion, *OR* odds ratio

Multiplicative interaction analysis showed that WML volume did not significantly modify the effect of heparin on good functional outcome (*p* = 0.65), any ICH (*p* = 0.26), aICH (*p* = 0.08), and sICH (*p* = 0.63) (Table 3).

Both the sensitivity analyses adjusted for treatment with IVT and the one adjusted for prior use of antithrombotics showed similar results as the main analysis (Appendix 3 and 4).

## Discussion

This post-hoc analysis of the MR CLEAN-MED trial suggests that WML volume significantly modifies the effect of aspirin on sICH during EVT. The aspirin-associated sICH risk decreased by 4% per 1 mL increase in WML volume and became non-significant above 11.35 mL, suggesting that the relationship between periprocedural aspirin and sICH risk is not uniform across all patients but may depend on underlying small vessel disease burden. The effect of aspirin on good functional outcome, any ICH, and aICH was not modified by WML volume. Similarly, the effect of heparin on good functional outcome and ICHs was not modified by WML volume.

The aspirin-sICH effect modification contradicts our initial hypothesis, suggesting complex interactions between WML pathology and aspirin mechanisms that warrant further investigation. While we cannot definitively explain this interaction, it may suggest that in patients with larger WML volumes, pathological mechanisms like compromised blood-brain barrier integrity [12–14], and impaired microvascular reactivity and integrity [1, 12, 13, 15–18] elevate baseline sICH risk. The proportional increase in sICH risk attributable to aspirin might be smaller in these patients compared to those with minimal WML burden, indicating non-additive interaction where aspirin’s impact diminishes as WML-related vascular pathology increases. Alternative explanations should also be considered. Patients with larger WMLs might have experienced severe impairment from stroke such that hemorrhagic transformation did not cause additional clinical deterioration, resulting in classification as aICH rather than sICH. However, we found no evidence of increased aICH or decreased sICH in aspirin-treated patients with severe strokes and large WML volumes, making this explanation unlikely. Another explanation involves aspirin’s antiplatelet effects, potentially countering specific WML-related processes that elevate sICH risk. These processes include (i) platelet hyperactivation and hypercoagulability [19, 20], which reduce tissue perfusion, and (ii) decreased microvascular reactivity and vessel narrowing [15], which increase the risk of microcirculation occlusions. Aspirin might mitigate these effects by counteracting platelet hyperactivation and reducing microthrombi formation. However, if truly protective, we would expect lower sICH rates in aspirin-treated patients with large WML volumes, yet both treated and untreated groups showed identical rates (16%). Finally, with the sensitivity analyses, we ruled out IVT and prior use of antithrombotics as confounders, which are both strongly associated with increased hemorrhage risk [21].

Regarding functional outcome, larger WML volumes were associated with worse mRS scores, in line with previous studies [3–5, 22–25]. We extended these findings by showing that this association persisted independently of aspirin and heparin treatment, which could be supported by the finding that aspirin and heparin use themselves were not associated with worse functional outcomes, consistent with the MR CLEAN-MED results [6]. This WML-outcome relationship likely reflects multiple mechanisms: reduced ischemic resilience due to small vessel disease, which impairs microvascular reactivity and integrity [1, 12, 13, 15–18], hindered post-stroke recovery from disrupted network connectivity and decreased neuronal plasticity [2, 26–28], reduced tissue perfusion from ongoing platelet activation and hypercoagulability [19, 20], bleeding complications from blood-brain barrier damage [12–14], WML-associated health impairments interfering with stroke recovery [25, 29–33], and the cardiovascular risk factor burden associated with WMLs. However, these results should be interpreted cautiously, as the strong correlation between age and WMLs creates potential for residual confounding despite our adjustment efforts.

For hemorrhagic complications, findings in the literature are contradictory regarding sICH and WML burden. While systematic reviews and meta-analyses show an association between increased WML burden and higher sICH risk following IVT [3, 4], WML burden did not seem to affect sICH occurrence in EVT-treated populations [5, 23, 24]. We observed higher sICH incidence in patients with larger WML volumes (tertile 3: 16% vs tertile 1–2: 7–8%), but multivariate analysis showed no statistically significant independent association after adjustment. This discrepancy may result from the aspirin- and heparin-induced sICH risk overshadowing WML volume’s contribution in our models. The control arm (patients allocated to receive neither aspirin nor heparin) showed an increasing trend in sICH rates across tertiles (2%, 2%, and 11%, respectively), suggesting a potential WML-sICH association independent of aspirin and heparin, though the low incidence precludes definitive conclusions. Regarding any ICH and aICH, limited research exists on their association with WMLs, and previous studies found no significant association between WML volume and aICH [8] or hemorrhagic transformation [22], consistent with our findings.

Our assessment of WML-outcome associations may differ from previous literature due to our use of NCCT rather than MRI. Furthermore, following recommended practice for effect modification studies [11], we adjusted only for confounders related to aspirin and heparin treatment, aligning with MR CLEAN-MED variables rather than adjusting for WML-outcome confounders used in various previous studies. However, given that these studies adjusted for their own distinct sets of variables, the overall comparability is inherently limited.

We analyzed the intention-to-treat population to study how WML-based individualized effects of aspirin and heparin would occur in clinical practice, preserving randomization benefits, though we acknowledge that complementary analyses, such as per-protocol, could have provided additional insights into the direct impact of the treatments.

Our study has several limitations. First, any conclusions regarding effect modification on sICH should be interpreted cautiously, given its low incidence. Second, potential selection bias from trial criteria and the requirement for baseline NCCT may limit generalizability to clinical practice. Third, as this is a post-hoc analysis with data-driven thresholds, there’s an increased risk of Type I errors, making the observed aspirin effect modification hypothesis-generating rather than definitive. Fourth, the 11.35 mL WML volume threshold was statistically derived rather than based on established clinical criteria, and with most patients having volumes below this threshold, findings for larger volumes warrant caution due to the smaller sample size. Additional limitations stem from the parent MR CLEAN-MED trial: precision limitations from early termination (though unlikely to have affected the adverse outcome findings); potential bias in post-intervention care due to unblinded treatment allocation (though imaging and outcomes were independently assessed); lack of recording for heparin use in pressure bags; high percentage of transferred patients from a primary hospital; and potential selection bias from deferred consent (though safety register analysis showed comparable effect estimates).

The unexpected attenuation of aspirin-associated sICH risk with increasing WML volume requires validation in independent cohorts. Future studies should explore the underlying pathophysiological mechanisms driving this interaction in larger populations. Research should investigate whether this effect modification represents a true protective mechanism or reflects methodological artifacts such as competing risks or ceiling effects in high-risk patients. Additionally, studies should examine whether similar effect modifications exist for other antithrombotic agents and in different clinical scenarios, such as acute stenting procedures.

In conclusion, WML volume modified the effect of intravenous aspirin during EVT on sICH, with aspirin-associated sICH risk decreasing as WML volume increased. No additional sICH risk from aspirin was observed in patients with large WML volumes. WML volume did not modify the effect of aspirin and heparin on functional outcome, any ICH and aICH, nor heparin’s effect on sICH.

## Supplementary information

ELECTRONIC SUPPLEMENTARY MATERIAL

## Data Availability

Source data will not be shared, as patient consent for releasing anonymized data was not obtained. Nevertheless, detailed analytical methods and study materials, including statistical analysis output files, will be made available to researchers upon request to the corresponding author.

## Abbreviations

AIS: Acute ischemic stroke
ASPECTS: Alberta Stroke Program Early CT score
CI: Confidence interval
EVT: Endovascular thrombectomy
IVT: Intravenous thrombolysis
LVO: Large vessel occlusion
mRS: Modified Rankin scale
NCCT: Non-contrast computed tomography
NIHSS: National Institutes of Health Stroke Scale
sICH: Symptomatic intracranial hemorrhage
WML: White matter lesion
