# Supplementary material for: White matter lesion effect modification of aspirin and unfractionated heparin during endovascular stroke treatment
Source: Insights Imaging. 2025 Oct 22;16:224. doi: 10.1186/s13244-025-02095-2 (PMC12546164; doi:10.1186/s13244-025-02095-2)
Supplement: Supplementary file 1 — ELECTRONIC SUPPLEMENTARY MATERIAL [file 13244_2025_2095_MOESM1_ESM.pdf]

White Matter Lesion Effect Modification of Aspirin and Unfractionated Heparin during Endovascular Stroke Treatment

ELECTRONIC SUPPLEMENTARY MATERIAL

Appendix 1 – STROBE Statement

	Item No	Recommendation	Page No
**Title and abstract**	1	(*a*) Indicate the study's design with a commonly used term in the title or the abstract	1
		(*b*) Provide in the abstract an informative and balanced summary of what was done and what was found	1
**Introduction**			
Background/ rationale	2	Explain the scientific background and rationale for the investigation being reported	3
Objectives	3	State specific objectives, including any prespecified hypotheses	3-4
**Methods**			
Study design	4	Present key elements of study design early in the paper	4
Setting	5	Describe the setting, locations, and relevant dates, including periods of recruitment, exposure, follow-up, and data collection	4
Participants	6	(*a*) *Cohort study*—Give the eligibility criteria, and the sources and methods of selection of participants. Describe methods of follow-up<br>*Case-control study*—Give the eligibility criteria, and the sources and methods of case ascertainment and control selection. Give the rationale for the choice of cases and controls<br>*Cross-sectional study*—Give the eligibility criteria, and the sources and methods of selection of participants	4
Variables	7	Clearly define all outcomes, exposures, predictors, potential confounders, and effect modifiers. Give diagnostic criteria, if applicable	4-5
Data sources/ measurement	8*	For each variable of interest, give sources of data and details of methods of assessment (measurement). Describe comparability of assessment methods if there is more than one group	4-5
Bias	9	Describe any efforts to address potential sources of bias	5-6, 9-11
Study size	10	Explain how the study size was arrived at	4, Fig. A
Quantitative variables	11	Explain how quantitative variables were handled in the analyses. If applicable, describe which groupings were chosen and why	4-5
Statistical methods	12	(*a*) Describe all statistical methods, including those used to control for confounding	5-6
		(*b*) Describe any methods used to examine subgroups and interactions	5-6
		(*c*) Explain how missing data were addressed	6
		(*e*) Describe any sensitivity analyses	6

Results

Participants	13*	(a) Report numbers of individuals at each stage of study—eg numbers potentially eligible, examined for eligibility, confirmed eligible, included in the study, completing follow-up, and analysed	6, Fig. A
		(b) Give reasons for non-participation at each stage	6, Fig. A
		(c) Consider use of a flow diagram	Fig. A
Descriptive data	14*	(a) Give characteristics of study participants (eg demographic, clinical, social) and information on exposures and potential confounders	6-7 T1
		(b) Indicate number of participants with missing data for each variable of interest	T1
		(c) *Cohort study*—Summarise follow-up time (eg, average and total amount)	
Outcome data	15*	*Cohort study*—Report numbers of outcome events or summary measures over time<br>*Case-control study*—Report numbers in each exposure category, or summary measures of exposure<br>*Cross-sectional study*—Report numbers of outcome events or summary measures	T1
Main results	16	(*a*) Give unadjusted estimates and, if applicable, confounder-adjusted estimates and their precision (eg, 95% confidence interval). Make clear which confounders were adjusted for and why they were included	7-8 T2-3
		(*b*) Report category boundaries when continuous variables were categorized	T1
		(*c*) If relevant, consider translating estimates of relative risk into absolute risk for a meaningful time period	
Other analyses	17	Report other analyses done—eg analyses of subgroups and interactions, and sensitivity analyses	7-8
**Discussion**			
Key results	18	Summarise key results with reference to study objectives	8
Limitations	19	Discuss limitations of the study, taking into account sources of potential bias or imprecision. Discuss both direction and magnitude of any potential bias	10-11
Interpretation	20	Give a cautious overall interpretation of results considering objectives, limitations, multiplicity of analyses, results from similar studies, and other relevant evidence	8-10
Generalisability	21	Discuss the generalisability (external validity) of the study results	10-11
**Other information**			
Funding	22	Give the source of funding and the role of the funders for the present study and, if applicable, for the original study on which the present article is based	11-12

Appendix 2 – Supplemental Methods; Model Adjustments

Model Architecture and Training Approach

We developed an automated WML segmentation model using nnU-Net, an open-source, out-of-the-box deep learning framework specifically designed for biomedical image segmentation[1]. Building upon our previous study[2], we implemented several key improvements to enhance segmentation accuracy and clinical applicability.

The training dataset consisted of NCCTs from Imperial College London (ICL), maintaining the same train-test split as our previous work (100 training, 20 test cases), stratified by WML volume to ensure balanced representation across severity levels. Three expert neuroradiologists independently performed manual WML segmentations on these NCCT scans, providing multiple ground truth annotations for each case.

To address the challenge of inter-rater variability commonly observed in WML assessment, we adopted a novel multi-label training approach. Instead of using a single consensus segmentation generated through the STAPLER algorithm[3], we provided the model with all three expert annotations for each scan. This approach enabled the model to learn from the natural variability in expert interpretations, potentially improving generalization to real-world clinical scenarios where such variability exists.

Additionally, we augmented the training set with 50 NCCTs from the MR CLEAN Registry[4], a prospective multicenter observational study of acute ischemic stroke patients treated with EVT. These scans were specifically selected from patients determined to have no WMLs by expert assessment [BE], allowing the model to learn the appearance of healthy brain tissue and improve specificity by reducing false positive segmentations.

We transitioned from a 2-dimensional to a 3-dimensional segmentation approach, recognizing that WMLs represent volumetric pathology that spans multiple image slices. The 2D approach suffered from several limitations: (1) many training slices contained no WML pathology despite patients having WMLs elsewhere in the brain, creating an imbalanced learning scenario; (2) limited spatial context led to increased false positives in cortical and subcortical regions; and (3)

post-processing restrictions to a 10mm radius around the ventricles were required to manage these false positives, potentially excluding clinically relevant lesions. The 3D approach addressed these limitations by providing complete volumetric context, incorporating spatial relationships across adjacent slices, and enabling the inclusion of all WML segmentations without geometric restrictions.

Similar to our previous model, we utilized nnU-Net's default training settings with 5-fold cross-validation. The final model represents an ensemble of models trained across all folds, with predictions generated by averaging softmax probabilities based on Dice similarity coefficient optimization. Figure A illustrates the data flow for model development, validation, and clinical application.

Model Validation

We conducted validation to ensure model reliability and clinical relevance. Model performance was evaluated against ground truth labels from each of the three experts individually and against a consensus label generated using the STAPLER algorithm.

To validate our multi-label training approach, we compared two training strategies: (1) traditional training using STAPLER-generated consensus labels, and (2) our novel approach using multiple expert labels. Performance evaluation on the test set demonstrated superior Dice similarity coefficients for the multi-label trained model across all ground truth comparisons (Expert 1: 0.44, Expert 2: 0.43, Expert 3: 0.49, STAPLER: 0.53) compared to the single-label approach (Expert 1: 0.42, Expert 2: 0.40, Expert 3: 0.47, STAPLER: 0.51) (Table A).

To contextualize model performance, we assessed inter-rater agreement among the three expert annotators. Remarkably, our automated model achieved higher Dice similarity coefficients with individual experts than the experts achieved with each other, suggesting that the model successfully learned to navigate inter-rater variability while maintaining consistency across different annotation styles (Figure B).

We validated the clinical relevance of our automated WML volume measurements by correlating them with established visual rating scales. WML volumes showed expected increases

across Fazekas score categories (No lesions, Mild, Moderate, Severe), confirming that our quantitative measurements align with established clinical assessments (Figure C).

Model Application

For the MR CLEAN-MED analysis, we applied this model to automatically segment WMLs from baseline NCCT scans and calculate total WML volumes for all patients with available imaging. This approach provided objective, quantitative WML burden assessment suitable for statistical modeling of treatment effect modification.

References

1. Isensee F, Jaeger PF, Kohl SAA, Petersen J, Maier-Hein KH. nnU-Net: a self-configuring method for deep learning-based biomedical image segmentation. *Nature Methods*. 2021;18(2):203–211.

2. van Voorst H, Pitkanen J, van Poppel L, de Vries L, Mojtahedi M, Martou L, Emmer BJ, Roos YB, van Oostenbrugge R, Postma AA, et al. Deep learning-based white matter lesion volume on CT is associated with outcome after acute ischemic stroke. *European Radiology*. 2024;i:2003–2023.

3. Warfield SK, Zou KH, Wells WM. Validation of image segmentation and expert quality with an expectation-maximization algorithm. *Lecture Notes in Computer Science (including subseries Lecture Notes in Artificial Intelligence and Lecture Notes in Bioinformatics)*. 2002;2488:298–306.

4. Jansen IGH, Mulder MJHL, Goldhoorn RJB. Endovascular treatment for acute ischaemic stroke in routine clinical practice: Prospective, observational cohort study (MR CLEAN Registry). *BMJ (Online)*. 2018;360.

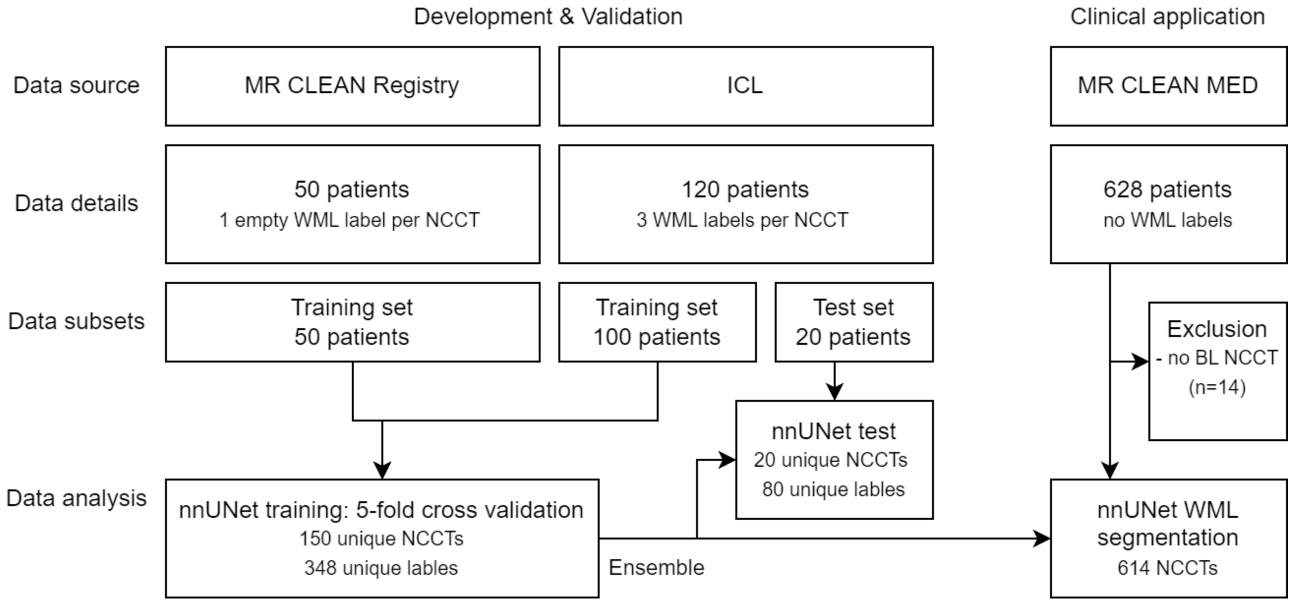

Figure A: Flow Chart of train, test, and application sets. BL=baseline. ICL=Imperial College Londen. MR CLEAN=Multicenter Randomized Clinical Trial of Endovascular Treatment for Acute Ischemic Stroke in the Netherlands. NCCT=non-contrast computed tomography. WML=white matter lesion.

Table A: Dice similarity coefficient comparison between stapler single-label and multi-label training approaches. Performance metrics show superior agreement of the multi-label trained nnU-Net model compared to the stapler single-label approach when evaluated against individual expert annotations and STAPLER consensus labels. Values represent Dice similarity coefficients on the test set.

	Model	
**Ground truth**	**Stapler single-label**	**Multi-label**
Expert 1	0.42	0.44
Expert 2	0.40	0.43
Expert 3	0.47	0.49
STAPLER	0.51	0.53

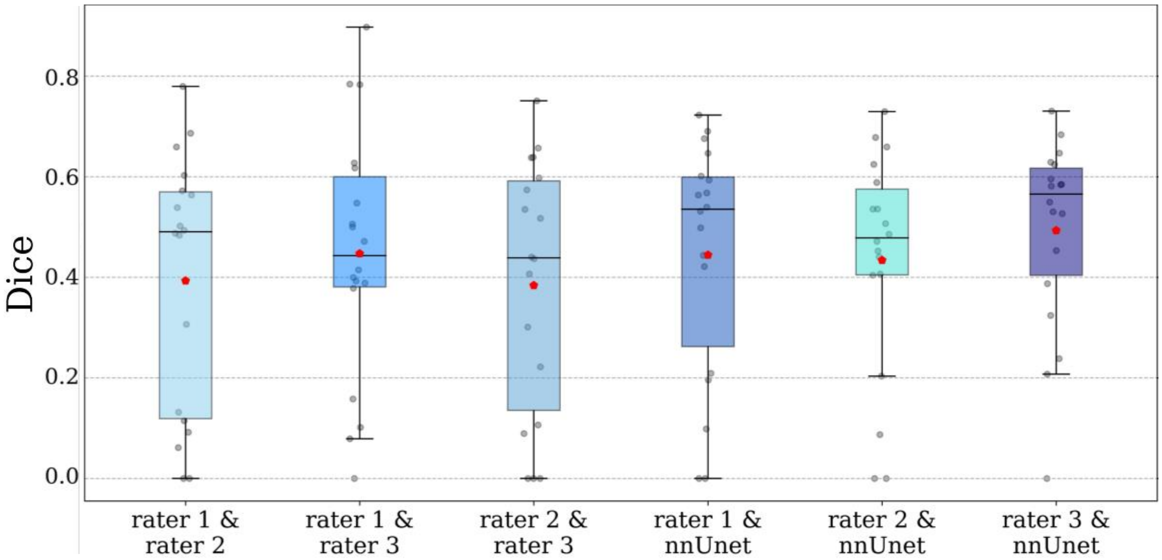

Figure B: Inter-rater agreement analysis comparing expert annotators and automated segmentation model. Boxplots show Dice similarity coefficients between different rater combinations and the nnU-Net model. The automated model achieved comparable or higher agreement with individual experts than the experts achieved with each other, demonstrating successful learning of annotation patterns across different expert styles. Red dots indicate mean values.

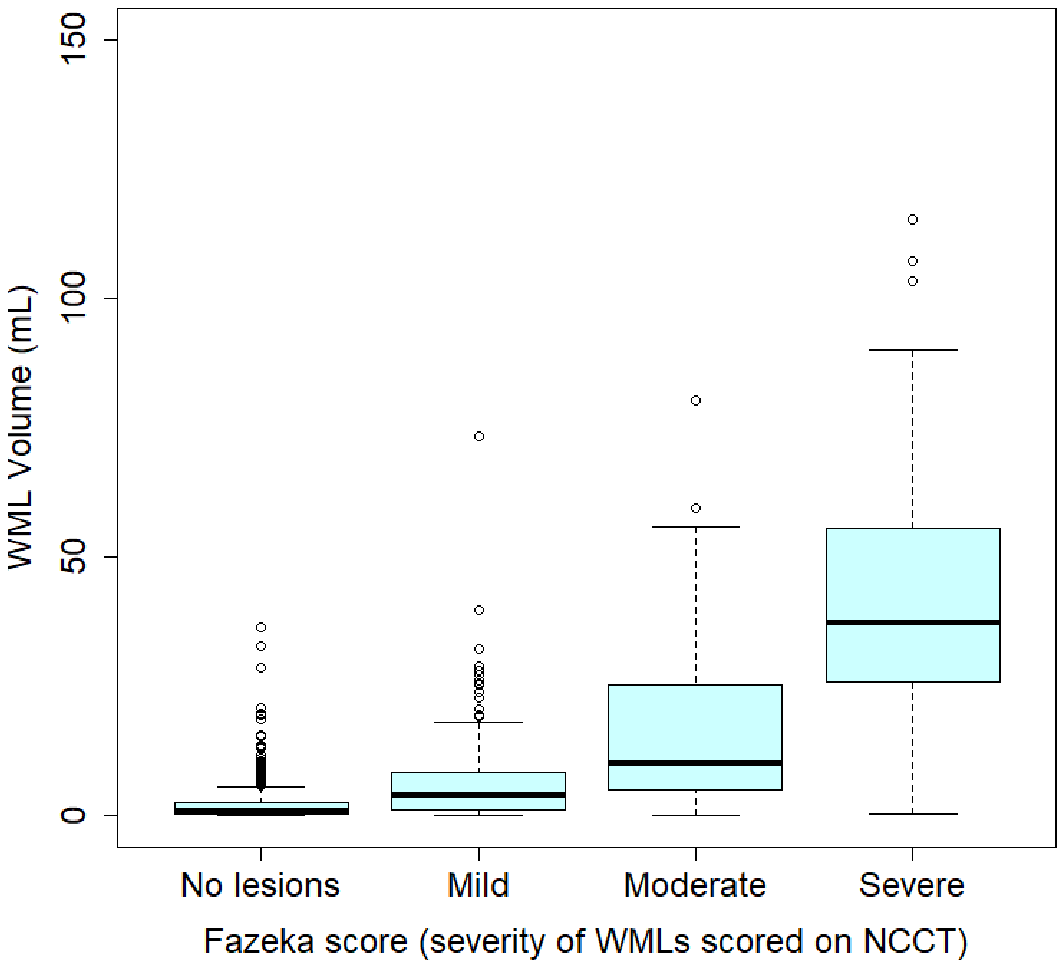

Figure C: WML volume distribution across Fazekas score categories. Box plots demonstrate increasing WML volumes from no lesions through severe categories on the Fazekas scale, validating the clinical relevance of automated volume measurements. The distribution confirms expected correlation between visual rating scales and quantitative volume assessments (severity of WMLs scored on NCCT). NCCT=non-contrast computed tomography. WML=white matter lesion.

Appendix 3 – Table B Sensitivity analyses with IVT

Table B: Treatment effects (95% CI) derived from the additive and multiplicative interaction models

		Additive model		Multiplicative interaction model		
**Outcome**		**Aspirin**	**WML volume**	**Aspirin**		**p-value**
		OR (95% CI)	OR (95% CI)	OR (95% CI) WML volume=0	OR (95% CI) interaction term	interaction
mRS score of 0-2 at 90 days	unadjusted	0.78 (0.57-1.07)	0.94 (0.92-0.96)	0.77 (0.53-1.12)	0.99 (0.95-1.03)	0.64
	adjusted*	0.79 (0.54-1.15)	0.96 (0.94-0.98)	0.80 (0.53-1.21)	1.00 (0.96-1.04)	0.93
any ICH	unadjusted	1.35 (0.98-1.85)	1.01 (1.00-1.02)	1.36 (0.96-1.94)	1.00 (0.98-1.02)	0.91
	adjusted*	1.36 (0.98-1.90)	1.00 (0.99-1.01)	1.43 (0.99-2.06)	0.99 (0.97-1.02)	0.55
aICH	unadjusted	1.04 (0.75-1.45)	1.00 (0.99-1.01)	0.92 (0.64-1.33)	1.02 (1.00-1.04)	0.12
	adjusted*	1.08 (0.76-1.52)	1.00 (0.98-1.01)	0.98 (0.67-1.44)	1.01 (0.99-1.04)	0.33
sICH	unadjusted	2.01 (1.18-3.44)	1.01 (1.00-1.03)	2.95 (1.57-5.52)	0.96 (0.93-0.99)	0.01
	adjusted*	1.89 (1.09-3.28)	1.01 (0.99-1.02)	2.79 (1.46-5.32)	0.96 (0.93-0.99)	0.01

**Outcome**		**Heparin**	**WML volume**	**Heparin**		**p-value**
		OR (95% CI)	OR (95% CI)	OR (95% CI) WML volume=0	OR (95% CI) interaction term	interaction
mRS score of 0-2 at 90 days	unadjusted	0.73 (0.53-1.00)	0.94 (0.92-0.96)	0.83 (0.57-1.20)	0.96 (0.92-1.01)	0.12
	adjusted*	0.77 (0.53-1.13)	0.96 (0.94-0.98)	0.80 (0.53-1.22)	0.99 (0.95-1.03)	0.70
any ICH	unadjusted	1.19 (0.87-1.64)	1.01 (1.00-1.02)	1.24 (0.87-1.76)	0.99 (0.97-1.02)	0.58
	adjusted*	1.11 (0.79-1.55)	1.00 (0.99-1.01)	1.21 (0.84-1.75)	0.99 (0.96-1.01)	0.27
aICH	unadjusted	0.95 (0.68-1.32)	1.00 (0.99-1.01)	1.06 (0.73-1.53)	0.98 (0.96-1.01)	0.18
	adjusted*	0.87 (0.61-1.23)	1.00 (0.98-1.01)	1.00 (0.68-1.47)	0.98 (0.96-1.00)	0.10
sICH	unadjusted	1.84 (1.07-3.15)	1.01 (1.00-1.03)	1.63 (0.89-2.98)	1.01 (0.98-1.05)	0.47
	adjusted*	1.86 (1.06-3.25)	1.00 (0.99-1.02)	1.75 (0.94-3.27)	1.01 (0.97-1.05)	0.73

**Values were adjusted for treatment with intravenous thrombolysis, age, pre-stroke mRS score, NIHSS at baseline, collateral score at baseline, time from onset to door of intervention hospital, time from door intervention hospital to groin puncture, and inclusion before or after early termination of moderate-dose unfractionated heparin arms. Effect estimates are quantified with the OR. mRS=modified Rankin Scale. sICH=symptomatic intracranial hemorrhage. WML=white matter lesion. OR=odds ratio. CI=confidence interval. NIHSS= National Institutes of Health Stroke Scale.*

Appendix 4 – Table C Sensitivity analyses with prior use of antithrombotics

Table C: Treatment effects (95% CI) derived from the additive and multiplicative interaction models

		Additive model		Multiplicative interaction model		
**Outcome**		**Aspirin**	**WML volume**	**Aspirin**		**p-value**
		OR (95% CI)	OR (95% CI)	OR (95% CI) WML volume=0	OR (95% CI) interaction term	interaction
mRS score of 0-2 at 90 days	unadjusted	0.78 (0.57-1.07)	0.94 (0.92-0.96)	0.77 (0.53-1.12)	0.99 (0.95-1.03)	0.64
	adjusted*	0.80 (0.55-1.16)	0.96 (0.94-0.98)	0.79 (0.52-1.21)	1.00 (0.96-1.04)	0.94
any ICH	unadjusted	1.35 (0.98-1.85)	1.01 (1.00-1.02)	1.36 (0.96-1.94)	1.00 (0.98-1.02)	0.91
	adjusted*	1.36 (0.98-1.90)	1.00 (0.99-1.01)	1.43 (0.99-2.07)	0.99 (0.97-1.02)	0.52
aICH	unadjusted	1.04 (0.75-1.45)	1.00 (0.99-1.01)	0.92 (0.64-1.33)	1.02 (1.00-1.04)	0.12
	adjusted*	1.07 (0.76-1.51)	0.99 (0.97-1.01)	0.98 (0.67-1.44)	1.01 (0.99-1.04)	0.30
sICH	unadjusted	2.01 (1.18-3.44)	1.01 (1.00-1.03)	2.95 (1.57-5.52)	0.96 (0.93-0.99)	0.01
	adjusted*	1.90 (1.10-3.30)	1.02 (1.00-1.04)	2.84 (1.48-5.43)	0.96 (0.93-0.99)	0.01

**Outcome**		**Heparin**	**WML volume**	**Heparin**		**p-value**
		OR (95% CI)	OR (95% CI)	OR (95% CI) WML volume=0	OR (95% CI) interaction term	interaction
mRS score of 0-2 at 90 days	unadjusted	0.73 (0.53-1.00)	0.94 (0.92-0.96)	0.83 (0.57-1.20)	0.96 (0.92-1.01)	0.12
	adjusted*	0.77 (0.53-1.13)	0.97 (0.94-0.98)	0.80 (0.53-1.21)	0.99 (0.95-1.04)	0.76
any ICH	unadjusted	1.19 (0.87-1.64)	1.01 (1.00-1.02)	1.24 (0.87-1.76)	0.99 (0.97-1.02)	0.58
	adjusted*	1.11 (0.80-1.55)	1.01 (0.99-1.03)	1.21 (0.84-1.75)	0.99 (0.96-1.01)	0.26
aICH	unadjusted	0.95 (0.68-1.32)	1.00 (0.99-1.01)	1.06 (0.73-1.53)	0.98 (0.96-1.01)	0.18
	adjusted*	0.87 (0.61-1.23)	1.01 (0.99-1.03)	1.00 (0.68-1.47)	0.98 (0.95-1.00)	0.08
sICH	unadjusted	1.84 (1.07-3.15)	1.01 (1.00-1.03)	1.63 (0.89-2.98)	1.01 (0.98-1.05)	0.47
	adjusted*	1.88 (1.07-3.29)	1.00 (0.96-1.03)	1.73 (0.93-3.25)	1.01 (0.97-1.05)	0.63

**Values were adjusted for prior use of antithrombotics (antiplatelets, vitamin K antagonists, DOACs), age, pre-stroke mRS score, NIHSS at baseline, collateral score at baseline, time from onset to door of intervention hospital, time from door intervention hospital to groin puncture, and inclusion before or after early termination of moderate-dose unfractionated heparin arms. Effect estimates are quantified with the OR. mRS=modified Rankin Scale. sICH=symptomatic intracranial hemorrhage. WML=white matter lesion. OR=odds ratio. CI=confidence interval. NIHSS= National Institutes of Health Stroke Scale.*

Appendix 5 – MR CLEAN-MED and CONTRAST Collaborators

MR CLEAN-MED investigators

Principal investigators
Diederik Dippel (MD, PhD),[1] Aad van der Lugt (MD, PhD)[1]

Study coordinators
Wouter van der Steen (MD),[1] Rob van de Graaf (MD, PhD),[1] Bob Roozenbeek (MD, PhD)[1]

Local principal investigators
Bob Roozenbeek (MD, PhD),[1] Adriaan van Es (MD, PhD),[1,2] Pieter Jan van Doormaal (MD),[1] Jonathan M. Coutinho (MD, PhD),[3] Bart Emmer (MD, PhD),[3] Inger de Ridder (MD, PhD),[4] Wim van Zwam (MD, PhD),[4] Bart van der Worp (MD, PhD),[5] Irene van der Schaaf (MD, PhD),[5] Rob Gons (MD, PhD),[6] Lonneke Yo (MD),[6] Jelis Boiten (MD, PhD),[7] Ido van den Wijngaard (MD, PhD),[2,7] Jeanette Hofmeijer (MD, PhD),[8] Jasper Martens (MD),[8] Wouter Schonewille (MD, PhD),[9] Jan Albert Vos, (MD, PhD),[9] Anil Man Tuladhar (MD, PhD),[10] Sjoerd Jenniskens (MD),[10] Karlijn de Laat (MD, PhD),[11] Lukas van Dijk (MD, PhD),[11] Heleen den Hertog (MD, PhD),[12] Boudewijn van Hasselt (MD),[12] Michel Remmers (MD),[13] Douwe Vos,[13] Anouk Rozeman (MD, PhD),[14] Otto Elgersma (MD, PhD),[14] Maarten Uyttenboogaart (MD, PhD),[15] Reinoud Bokkers (MD, PhD),[15] Julia van Tuijl (MD, PhD),[16] Issam Boukrab (MD),[16] Benjamin Gory (MD, PhD),[17] Arturo Consoli (MD),[18] Mikaël Mazighi (MD, PhD),[19] Frederic Clarencon (MD, PhD),[20] Gaultier Marnat (MD),[21]

Executive and writing committee
Diederik Dippel (MD, PhD),[1] Aad van der Lugt (MD, PhD),[1] Rob van de Graaf (MD, PhD),[1] Wouter van der Steen (MD),[1] Bob Roozenbeek (MD, PhD),[1] Adriaan van Es (MD, PhD),[1] Yvo Roos (MD, PhD),[3] Charles Majoie (MD, PhD),[3] Robert van Oostenbrugge (MD, PhD),[4] Wim van Zwam (MD, PhD),[4] Julie Staals (MD, PhD),[4] Sjoerd Jenniskens (MD),[10] Lukas van Dijk (MD, PhD),[11] Heleen den Hertog (MD, PhD),[12]

Local MR CLEAN-MED collaborators

Data Safety Monitoring Board
Peter Rothwell (MD, PhD) – *Chair,*[22] Andrew Molyneux (MD, PhD),[22] Joanna Moschandreas (MD, PhD)[22]

Independent trial statistician
Daan Nieboer (MSc)[1]

Advisory Board
Gregory del Zoppo (MD, PhD)[23]

CONTRAST clinical trial collaborators

Research leaders
Diederik Dippel (MD, PhD),[1] Charles Majoie (MD, PhD)[3]

Consortium coordinator:
Rick van Nuland, (PhD)[24]

Imaging assessment committee
Charles Majoie (MD, PhD) – *Chair*,[3] Aad van der Lugt (MD, PhD) – *Chair*,[1] Adriaan van Es, (MD, PhD),[1,2] Pieter-Jan van Doormaal (MD),[1] René van den Berg, (MD, PhD),[3] Ludo Beenen (MD),[3] Bart Emmer (MD, PhD),[3] Stefan Roosendaal (MD, PhD),[3] Wim van Zwam (MD, PhD),[4] Alida Annechien Postma (MD, PhD),[25] Lonneke Yo (MD, PhD),[6] Menno Krietemeijer (MD),[6] Geert Lycklama (MD, PhD),[7] Jasper Martens (MD),[8] Sebastiaan Hammer (MD, PhD),[10] Anton Meijer (MD, PhD),[10] Reinoud Bokkers (MD, PhD),[15] Anouk van der Hoorn (MD, PhD),[15] Ido van den Wijngaard (MD, PhD),[2,7] Albert Yoo (MD, PhD),[26] Dick Gerrits (MD)[27]

Adverse event committee
Robert van Oostenbrugge (MD, PhD) – *Chair*,[4] Bart Emmer (MD, PhD),[3] Jonathan M. Coutinho (MD, PhD),[3] Martine Truijman (MD, PhD),[4] Julie Staals (MD, PHD),[4] Bart van der Worp (MD, PhD),[5] J. Boogaarts (MD, PhD),[10] Ben Jansen (MD, PhD),[16] Sanne Zinkstok (MD, PhD)[28]

Outcome assessment committee
Yvo Roos (MD, PhD) – *Chair*,[3] Peter Koudstaal (MD, PhD),[1] Diederik Dippel (MD, PhD),[1] Jonathan M. Coutinho (MD, PhD),[3] Koos Keizer (MD, PhD),[5] Sanne Manschot (MD, PhD),[7] Jelis Boiten (MD, PhD),[7] Henk Kerkhoff (MD, PhD),[14] Ido van den Wijngaard (MD, PhD)[2,7]

Data management group
Hester Lingsma (PhD),[1] Diederik Dippel (MD, PhD),[1] Vicky Chalos (MD),[1] Olvert Berkhemer (MD, PhD)[1,3]

Imaging data management
Aad van der Lugt (MD, PhD),[1] Charles Majoie (MD, PhD),[3] Adriaan Versteeg,[1] Lennard Wolff (MD),[1] Matthijs van der Sluijs (MD),[1] Henk van Voorst (MD),[3] Manon Tolhuisen (MSc),[3]

Biomaterials and translational group
Hugo ten Cate (MD, PhD),[4] Moniek de Maat (PhD),[1] Samantha Donse-Donkel (MD),[1] Heleen van Beusekom (PhD),[1] Aladdin Taha (MD),[1] Aarazo Barakzie (MD)[1]

Local collaborators
Vicky Chalos (MD, PhD),[1] Rob van de Graaf (MD, PhD),[1] Wouter van der Steen (MD),[1] Aladdin Taha (MD),[1] Samantha Donse-Donkel (MD),[1] Lennard Wolff (MD),[1] Kilian Treurniet (MD),[3] Sophie van den Berg (MD),[3] Natalie LeCouffe (MD),[3] Manon Kappelhof (MD),[3] Rik Reinink (MD),[3] Manon Tolhuisen (MD),[3] Leon Rinkel (MD),[3] Josje Brouwer (MD),[3] Agnetha Bruggeman (MD),[3] Henk van Voorst (MD),[3] Robert-Jan Goldhoorn (MD),[4] Wouter Hinsenveld (MD),[4] Anne Pirson (MD),[4] Susan Olthuis (MD),[4] Simone Uniken Venema (MD),[5] Sjan Teeselink (MD),[10] Lotte Sondag (MD),[10] Sabine Collette (MD)[15]

Research nurses
Martin Sterrenberg,[1] Naziha El Ghannouti,[1] Laurine van der Steen,[3] Sabrina Verheesen,[4] Jeannique Vranken,[4] Ayla van Ahee,[5] Hester Bongenaar,[6] Maylee Smallegange,[6] Lida Tilet,[6] Joke de Meris,[7] Michelle Simons,[8] Wilma Pellikaan,[9] Wilma van Wijngaarden,[9] Kitty Blauwendraat,[9] Yvonne Drabbe,[11] Michelle Sandiman-Lefeber,[11] Anke Katthöfer,[11] Eva Ponjee,[12] Rieke Eilander,[12] Anja van Loon,[13] Karin Kraus,[13] Suze Kooij,[14] Annemarie Slotboom,[14] Marieke de Jong,[15] Friedus van der Minne,[15] Esther Santegoets[16]

Study monitors
Leontien Heiligers[1], Yvonne Martens,[1] Naziha El Ghannouti[1]

Affiliations

[1] Erasmus MC University Medical Center, Rotterdam, the Netherlands;
[2] Leiden University Medical Center, Leiden, the Netherlands;
[3] Amsterdam University Medical Centers, location AMC, Amsterdam, the Netherlands;
[4] Cardiovascular Research Institute Maastricht (CARIM), Maastricht University Medical Centre, Maastricht, The Netherlands;
[5] University Medical Center Utrecht, Brain Center Rudolf Magnus, Utrecht, the Netherlands;
[6] Catharina Hospital, Eindhoven, the Netherlands;
[7] Haaglanden Medical Centre, the Hague, the Netherlands;
[8] Rijnstate Hospital, Arnhem, the Netherlands;
[9] St. Antonius Hospital, Nieuwegein, the Netherlands;
[10] Radboud University Medical Center, Nijmegen, the Netherlands;
[11] HagaZiekenhuis, the Hague, the Netherlands;
[12] Isala, Zwolle, the Netherlands;
[13] Amphia Hospital, Breda, the Netherlands;
[14] Albert Schweitzer Hospital, Dordrecht, the Netherlands;
[15] University Medical Center Groningen, Groningen, the Netherlands;
[16] Elisabeth-TweeSteden Hospital, Tilburg, the Netherlands;
[17] University Hospital of Nancy, Nancy, France
[18] Foch Hospital, Suresnes, France
[19] Fondation Rothschild Hospital, Paris, France
[20] University Hospital of Bordeaux, Bordeaux, France
[21] Pitié-Salpêtrière University hospital, Paris, France
[22] John Radcliffe Hospital, Oxford, United Kingdom
[23] University of Washington, Seattle, Washington, United States
[24] Lygature, Utrecht, the Netherlands;
[25] School for Mental Health and Sciences (Mhens), Maastricht University Medical Center, Maastricht, The Netherlands
[26] Texas Stroke Institute, Dallas-Fort Worth, Texas, United States of America
[27] Medisch Spectrum Twente, Enschede, The Netherlands
[28] TerGooi, Hilversum, The Netherlands
